# *Vaccinium uliginosum* and *Vaccinium myrtillus*—Two Species—One Used as a Functional Food

**DOI:** 10.3390/nu15194119

**Published:** 2023-09-23

**Authors:** Agnieszka Kopystecka, Ilona Kozioł, Dominika Radomska, Krzysztof Bielawski, Anna Bielawska, Monika Wujec

**Affiliations:** 1 Students’ Scientific Circle on Medical Law at the Department of Humanities and Social Medicine, Medical University of Lublin, 20-093 Lublin, Poland; aga.kop@interia.eu (A.K.); ilona.koziol9@gmail.com (I.K.); 2 Department of Synthesis and Technology of Drugs, Faculty of Pharmacy, Medical University of Bialystok, Kilinskiego 1 Street, 15-089 Bialystok, Poland; dominika.radomska@sd.umb.edu.pl; 3 Department of Biotechnology, Faculty of Pharmacy, Medical University of Bialystok, Kilinskiego 1 Street, 15-089 Bialystok, Poland; anna.bielawska@umb.edu.pl; 4 Department of Organic Chemistry, Faculty of Pharmacy, Medical University of Lublin, 4a Chodzki Str., 20-093 Lublin, Poland

**Keywords:** bog bilberry, *Vaccinium uliginosum*, *Vaccinium myrtillus*, bilberry, bioactive compounds, bioactive natural products, dietary supplement

## Abstract

*Vaccinium uliginosum* L. (commonly known as bog bilberry) and *Vaccinium myrtillus* L. (commonly known as bilberry) are species of the genus *Vaccinium* (family *Ericaceae*). The red–purple–blue coloration of blueberries is attributed largely to the anthocyanins found in bilberries. Anthocyanins, known for their potent biological activity as antioxidants, have a significant involvement in the prophylaxis of cancer or other diseases, including those of metabolic origin. Bilberry is the most important economically wild berry in Northern Europe, and it is also extensively used in juice and food production. A review of the latest literature was performed to assess the composition and biological activity of *V. uliginosum* and *V. myrtillus*. Clinical studies confirm the benefits of *V. uliginosum* and *V. myrtillus* supplementation as part of a healthy diet. Because of their antioxidant, anti-inflammatory, anti-cancer, and apoptosis-reducing activity, both bog bilberries and bilberries can be used interchangeably as a dietary supplement with anti-free radical actions in the prevention of cancer diseases and cataracts, or as a component of sunscreen preparations.

## 1. Introduction

**Figure 1:**
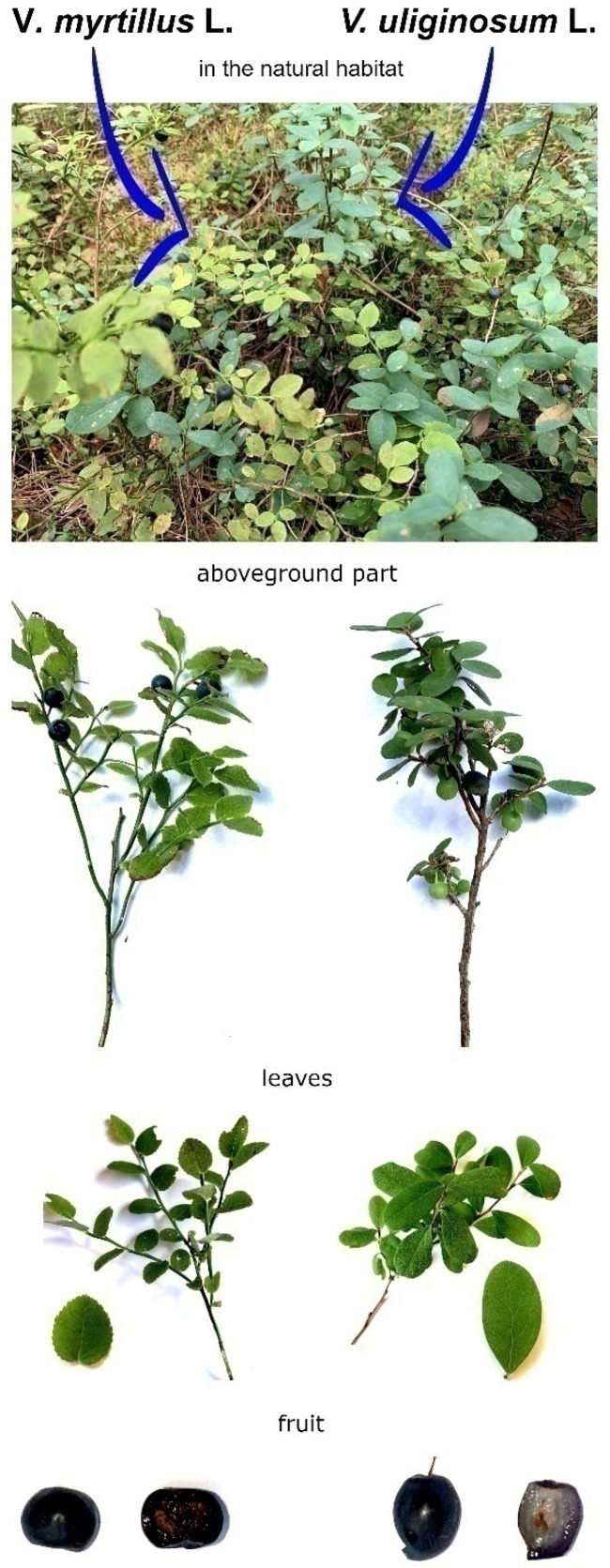
*V. myrtillus* and *V. uliginosum* in their natural habitat and the external appearance of their parts (leaves and fruit).

*Vaccinium uliginosum* L. (bog bilberry) and *Vaccinium myrtillus* L. (bilberry) are species of the genus *Vaccinium* (family *Ericaceae*). They are low-growing deciduous shrubs that produce dark purple fruits (berries) which are edible (Figure 1). Commonly called bilberries, their fruits are highly valued as a rich source of anthocyanins, which are naturally occurring compounds. In fresh berries, their content is about 0.5% [1,2,3,4]. In addition to fresh fruit, berries can also be consumed as frozen, dried, juices, jams, and food supplements [5]. It has recently become more popular to consume fermented products made from bilberries [6,7]. In vitro studies have shown that bilberry extracts have an impact on the effects of, among other things, anti-glycation and the scavenging of external radicals. Strong antioxidant properties were also found because of the occurrence of abundant bioactive substances, such as anthocyanins and flavanols [1,8,9]. Thanks to these properties, the supplementation of bilberries can have an impact on health in many cases of diseases. Its known pharmacological effects include vascular regulation, dysentery, antigens, diabetic retinopathy, and potential anti-cancer effects [10,11,12,13,14].

There are many studies on *Vaccinium* species, but so far there is no comparison of both species, *V. uliginosum* and *V. myrtillus*, especially in terms of their biological activity and possible use as functional food. The biological effect of the fruit extract of *V. uliginosum* is known primarily from both Chinese and European folk medicine. In the presented review, we want to present the similarities and differences between two species growing side by side in their natural habitat.

## 2. Occurrence

Most *V. myrtillus* and *V. uliginosum* are mainly acquired from their native habitats [15,16]. These members of the *Ericaceae* family grow best in humid and moderate climates. Mountains and high mountains are the most common habitats in their southernmost distribution [17]. *V. myrtillus* is found in European mountains and forests, while *V. uliginosum* grows in areas of Asia, Europe, and North America [18]. *V. uliginosum*, *V. myrtillus*, and *V. vitis-idaea* are the species that grow on the Iberian Peninsula. Observations of *V. uliginosum* on Portugal’s mainland suggest fragmented populations and uncertain survival in the uppermost parts of Serra da Estrela. Serra da Estrela as well as Serra da Freita both have fragmented populations of *V. myrtillus*, but the latter is more plentiful in northern Portugal’s mountains [19]. Bilberry (*Vaccinium myrtillus* L.) is the most important economically wild berry in Northern Europe, and it is also extensively used in juice and food production. The bog bilberry is used to a lesser extent, but it is widespread in northern areas [20]. Compared to cultivated species, wild berries have a more complex chemical composition [18]. A very important aspect is also climate and weather conditions, which determine the content of the various bioactive substances (phenolic acids, anthocyanins, etc.) in blueberries [21]. In turn, the qualitative–quantitative composition of phenolic compounds in bilberries depends on the plant parts used, growth stage, and genetic factors [22,23]. For this reason, buyers are interested in the origin of the berries, as those from specific areas or countries often have a higher price. As spectrophotometers are quick and easy to use, they are highly suitable for commercial purposes, especially for evaluating berry quality [24,25,26].

A study by Urbanaviciene and Dalia et al. determined the physicochemical properties, as well as the levels of total anthocyanins (TAC) and polyphenols (TPC) present in *V. myrtillus* populations, which occur in areas of Northern Europe (Lithuania, Latvia, Finland, and Norway), along with their ability to scavenge free radicals. In the investigation, *V. myrtillus* had pH values ranging from 2.94 to 3.47. Approximately 232.7 to 475.5 mg/100 g of fresh weight (FW) were obtained from the investigated *V. myrtillus* samples. The content of TPC was the highest in Norway and the lowest in Lithuania and varied between 452–902 mg/100 g FW. According to the study, the antioxidant capacity of *V. myrtillus* oscillated between 60.9 and 106.0 mol TE/g FW, with the lowest value in populations from Lithuania and the highest from Norway [27]. The main ingredients that make up more than 50% of the Lithuanian bilberry water extract are cyanidin-3-*O*-glucoside, cyanidin-3-*O*-arabinoside, delphinidin-3-*O*-galactoside, peonidin-3-*O*-glucoside, petunidin-3-*O*-glucoside, delphinidin glycosides, and cyanidin [28]. According to Szakiel et al., the content of triterpenoids in the leaves of *V. myrtillus* from wild habitats varies significantly depending on its location in Poland and Finland. Polish leaves were significantly richer in lupeol, and friedelin was only found on Finnish leaves, while taraxasterol was only found on leaves of plants from Poland. Polish leaves contained more than three times as much 2α-hydroxyursolic and 2α-hydroxyoleanolic acids as Finnish leaves, but they had similar levels of oleanolic and ursolic acids [29].

## 3. *V. uliginosum* and *V. myrtillus* Composition

Blueberry composition depends on the genotype of the plant [30,31,32]. *V. uliginosum* berries contain many anthocyanins and flavonols. *V. uliginosum* has a characteristic profile of flavonols and anthocyanins compared to other berries of the *Vaccinium* family, which can be used to distinguish bog bilberry from *V. myrtillus* [33]. *V. myrtillus* seeds and oils contain natural antioxidants, anti-inflammatory, anti-atherosclerotic, and anticancer compounds, such as tocochromanols, carotenoids, flavonoids, phytosterols, and phenolic acids [34,35]. The caloric energy intake of fresh bilberries is approximately 45 kcal/100 g. They consist of water (84%), carbohydrates (9.6%), proteins (0.7%), fats (0.4%), and fibers (about 3.5%) [36]. This is compared to dry bilberry, which has 395 kcal/100 g and contains 94% carbohydrates, 3% proteins, and 1.5% fats [37]. The pH value of the bog bilberry’s berry (*V. uliginosum* L.) was relatively high (pH = 3.5), and their titratable acidity, in turn, was moderate (1 g of citric acid/100 g). The main identified soluble sugar was fructose (concentration of 2138 ± 149 mg/100 g FW), while glucose was the second in amount (concentration of 1664 ± 121 mg/100 g FW) [38].

### 3.1. Polyphenols

**Figure 2:**
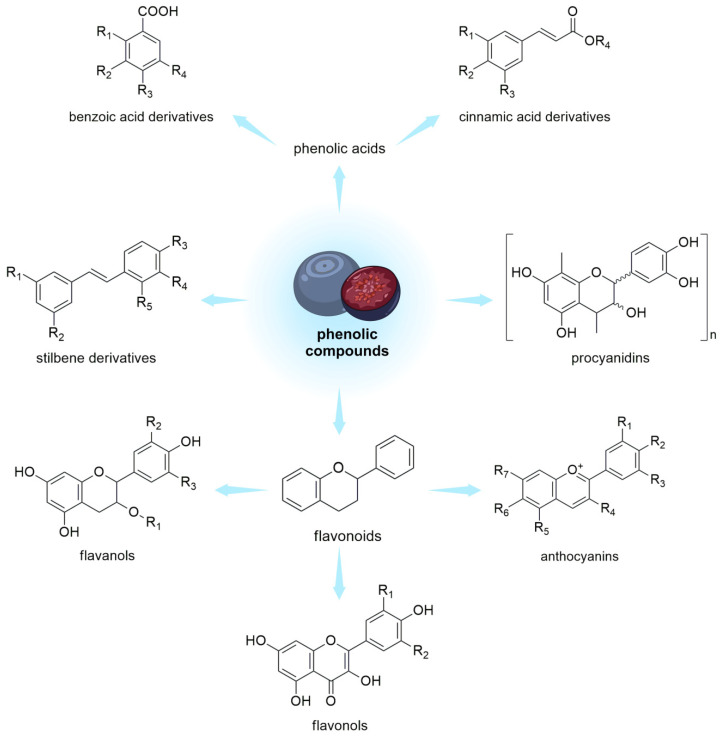
Phenolic compounds in *V. uliginosum* and *V. myrtillus*.

Polyphenols are a group of naturally occurring compounds found in various plant foods, including berries from the *Vaccinium* genus (Figure 2).

The content and availability of polyphenols in blueberries can be affected by various factors, including agricultural practices, storage, and processing technologies. Organic farming practices, which avoid synthetic pesticides and fertilizers, may promote higher polyphenol content in blueberries. This is because plants often produce more phytochemicals, including polyphenols, as a defense mechanism against pests and diseases. Harvesting techniques are very important too. Picking blueberries at the right ripeness can affect their polyphenol content. Polyphenol levels may increase as the berries ripen [39]. Using gentle harvesting methods to avoid damaging the berries can help preserve their polyphenol content. Proper temperature, pressure, and humidity control during storage are crucial to prevent polyphenol degradation [40]. Cold storage can help maintain polyphenol levels in fresh blueberries. Modified Atmosphere Packaging (MAP) involves adjusting the gas composition inside the packaging to extend the shelf life of blueberries while preserving their polyphenols [41]. Processing technology conditions are the most important factors influencing the content of polyphenols in products made from berries. Freeze drying is a method that can preserve the polyphenol content in blueberries by removing moisture without significant heat exposure, which can degrade polyphenols [42]. Drying blueberries at lower temperatures can help retain their polyphenol content compared to high-temperature drying methods. Processing blueberries into purees or juices can concentrate polyphenols. However, some heat exposure during processing may cause a slight reduction in polyphenol levels. Changes in the phenolic composition of berries may be related to various treatments, including ozone pretreatment using ultrasound [43] or using cold plasma [44].

Conventional methods for polyphenol extraction have limitations and drawbacks, which can include the use of harsh solvents, high energy consumption, and potential degradation of the polyphenols. These drawbacks have led to a growing demand for more sustainable and eco-friendly extraction techniques. To maximize the efficiency of polyphenol extraction while maintaining the total polyphenol content (TPC) and antioxidant capacity of the extract, it is essential to assess and compare different extraction conditions. Some novel technologies such as an ultrasound, microwave, cold plasma, pulsed electric field, and pressurized liquid were used as alternatives assisting the extraction process [45]. Factors such as temperature, pressure, and processing time can significantly influence the outcome.

It is important to note that while these technologies and practices can influence polyphenol content, the specific impact may vary depending on factors such as the blueberry variety and environmental conditions.

Polyphenol compounds in berries of *Vaccinium* spp. were determined by different methods (Table 1).

Quercetin, kaempferol, phenolic acid, and gentisic acid were the largest fraction of polyphenols identified in *V. myrtillus* extracts [54]. In one of the studies on *V. uliginosum gaultherioides* and *V. myrtillus* berries, differences in terms of relative percentages of total monomeric anthocyanins (TMA) concerning total soluble polyphenols (TSP) were shown, which was the predominant polyphenolic class in blueberry, but this was not observed in bog bilberry [55]. The bog bilberry juice was abundant in myricetin-3-*O*-galactoside and quercetin-3-*O*-galactoside [56]. The ferric reducing antioxidant power (FRAP) test yielded the highest antioxidant capacity values (117 μmol TE/g FW), followed by the oxygen radical absorbance capacity (ORAC) test (84 μmol TE/g FW) [38]. In a study by Wang Yu et al. in 10 different populations of *V. uliginosum* from the Changbai Mountains (China), the content of TF (total flavonoids), TA (total anthocyanins), and TP (total phenols) was assessed, and the spatial distribution and correlation between these components were examined. Fifteen anthocyanins were identified and described, and the amount of malvidin-glucoside, petunidin-glucoside, and delphinidin-glucoside was the highest in this phytochemical group. TF, TA, and TP values were the highest in the Dongfanghong forest farm (DFHI) and the Lanjia forest farm (LJII) populations, respectively. As compared to the other samples, the TF content of the DFHI-8 sample was higher, as was the TA content of the LJIII-1 and the TP content of the LJIII-4. At an altitude from 740 to 838 m, TA and TP content exhibited a positive correlation. In turn, at altitudes >838 m, their dependence showed negative values [57].

Antioxidant properties of juices of bog blueberry (*Vaccinium uliginosum*) were evaluated by ABTS scavenging capacity (RSC), FRAP, ORAC, TPC (total phenolic content), and TAC (total anthocyanin content) assays. The TPC values ranged from 0.85 to 2.81 mg gallic acid equivalent/mL; ORAC, FRAP, and RSC values were 4.21–45.68, 3.07–17.8, and 6.38–20.9 μmol Trolox equivalent/g, respectively. Bog blueberry had a very high TAC, 14.19 mg/100 mL. In the ABTS decolorization test, blueberry juices showed the highest RSC (20.9 μmol TE/g), FRAP (31.99 μmol Fe^2+^/g and 17.80 μmol TE/g), and ORAC (45.68 μmol TE/g). Bog bilberry, even though it contained moderate amounts of quantified compounds, showed a very high antioxidant capacity; it had a slightly different chromatographic profile. It was found that there was a moderate negative correlation between berry weight and both FRAP and ORAC assays. Berries with a larger mass probably accumulate more macronutrients, e.g., carbohydrates. The values obtained in the FRAP and ORAC assays also correlated with quinic and chlorogenic acid concentrations (*p* ≤ 0.01). According to the results of this study, new cultivars exhibiting higher antioxidant capacity can potentially be created through the use of the germplasm of half-highbush blueberry and *V. uliginosum* [58].

The team of Bayazid AB et al. conducted in vitro studies evaluating the antioxidant and anti-inflammatory properties of 70% ethanolic extracts of bilberry. Antioxidant activity was measured by total phenols, flavonoids, and ascorbic acid. Bilberry extract dose-dependently inhibited linoleic acid oxidation and showed free radical elimination activity. This extract reversed pro-inflammatory cytokines such as inducible nitric oxide synthase (iNOS), cyclooxygenase 2 (COX-2), tumor necrosis factor α (TNF-α), and interleukin-6 (IL-6) in LPS (lipopolysaccharide)-induced RAW 264.7 cells and suppressed NO (nitric oxide) generation. It was suggested that *V. myrtillus* blueberry extract is a natural preparation with strong antioxidant properties and acts as an anti-inflammatory agent due to its high concentration of anthocyanins [59].

#### 3.1.1. Flavonols and Flavanols

Latti et al., in their studies, were the first to show the presence of kaempferol and isorhamnetin aglycones in *V. uliginosum*. In their study, about 1/4 of bog blueberry samples contained more flavonols than anthocyanins [33].

**Figure 3:**
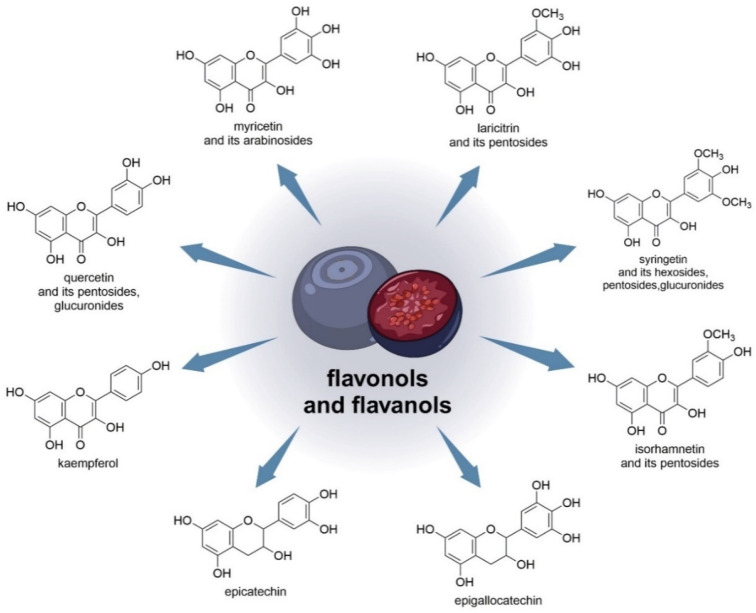
Chemical structures of flavonols and flavanols (and the sugars to which they are linked) that are contained in *V. uliginosum* and *V. myrtillus*.

One study found that EC (epicatechin) and EGC (epigallocatechin) were the major flavanols in blueberry juice [56]. *V. uliginosum* also contains flavonols such as laricitrin, syringetin, myricetin, and quercetin. Based on the findings of myricetin and quercetin arabinosides, the minor laricitrin, isorhamnetin, and syringing pentosides were further named arabinosides. Both laricitrin and isorhamnetin were also detected in *V. myrtillus* [33]. Laricithrin, isorhamnetin, myricetin, kaempferol, syringetinhexosides, pentosides, and glucuronides, as well as glucuronide and pentosides QUE (quercetin), were identified in bilberry and bog bilberry in various amounts, and flavonol predominance in bog bilberry [55]. Figure 3 shows the chemical structures of flavonols and flavanols (and the sugars to which they are linked) that are contained in *V. uliginosum* and *V. myrtillus*.

Due to the very high concentration of quercetin-3-galactoside, the prevalence of quercetin-3-rhamnoside in blueberries contrasts with QUE and its derivatives in *V. uliginosum* subsp. *gaultheroides*. Blueberries contained about ten times more QUE-3-RHA than bog blueberries [55].

#### 3.1.2. Anthocyanins

Compared with some common edible berries, bog bilberries contain more complex anthocyanins [60]. It was found that the TAC in blueberries is about 6 g/kg of fruit [61]. Holkem et al. researched *V. myrtillus* extracts. It was proven that the best antiproliferative effect was shown by an anthocyanin-rich extract due to the abundance of bioactive substances occurring in it; for this extract, there was an elevation in the antioxidative effect after the introduction of bacteria [62]. In one study conducted on *V. myrtillus* juice, results indicated that blueberry juice and cyanidin increased mitochondrial activity and reduced intracellular reactive oxygen species (ROS) generation and hydrogen peroxide-induced lipid peroxidation. In addition, the juice caused an increase in the activity of antiradical enzymes—superoxide dismutase (SOD) and catalase (CAT) [63]. It has been proven that they have antioxidant, anti-cancer, and anti-inflammatory effects and that these compounds can alleviate chronic and acute colitis [64,65]. Bog bilberries were the subject of one study that identified five key anthocyanidins, among which malvidin 3-glucoside was the main compound. It was observed that the TAC fraction showed particularly high variability in antioxidant capacity, which was mainly influenced by the type of phenolic structure that was eluted by solid-phase extraction (SPE) [38].

**Figure 4:**
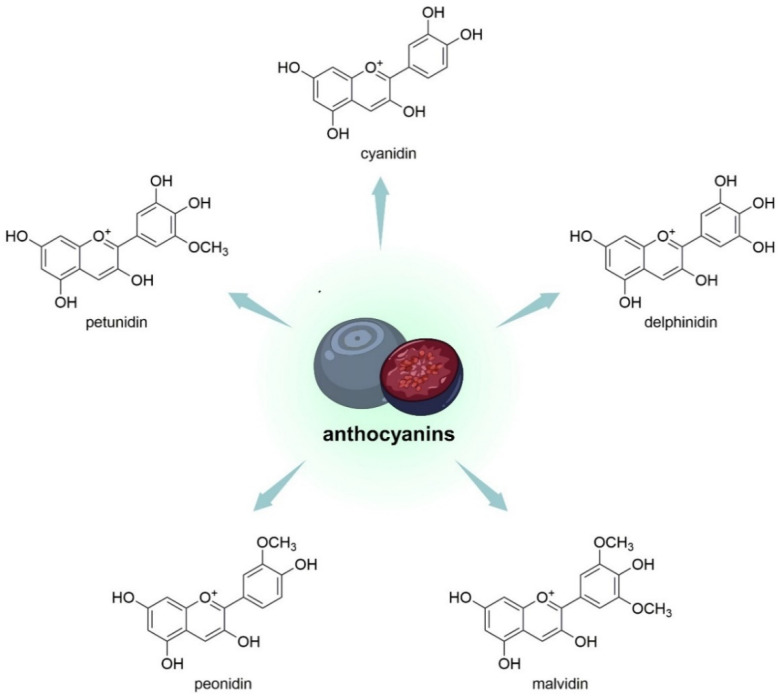
Chemical structures of aglycone parts of anthocyanins (so-called anthocyanidins) that are contained in *V. uliginosum* and *V. myrtillus*.

*V. uliginosum,* in their structure, have an aglycone part and a glycosyl part. Malvidin, delphinidin, cyanidin, peonidin, and petunidin constitute the aglycone part (Figure 4), while arabinoside, glucoside, xyloside and galactoside belong to the glycosyl part of the compound. In the course of the research, it was observed that specific habitats conditioned noticeable differences in the quantitative composition of anthocyanidin glycosides [33]. Most anthocyanins in *V. uliginosum* derived from B-ring tri-substituted anthocyanidins (80 ± 3%); the most important was the malvidin-type (46 ± 6%), followed by cyanidin (21 ± 3%), delphinidin (13 ± 3%), petunidin (13 ± 0%), and peonidin (7 ± 1%) [38].

*V. myrtillus* anthocyanin extracts contain at least 16 anthocyanin monomers [66]. In its extract composition were cyanidin-3-*O*-rutinoside, delphinidin-3-galactoside, delphinidin-3-glucoside, cyanidin-3-galactoside, and chlorogenic acid as the main native phenolic compounds. They were also contained in the extract in smaller amounts of petunidin-3-glucoside, malvidin-3-glucoside, and cyanidin-3-glucoside. Cyanidin-3-*O*-rutinoside was higher in *V. uliginosum* compared to *V. myrtillus,* accounting for 136.8909 mg/g ± 11.48 (36.63%) and 43.5743 mg/g ± 4.01 (26.40%) of total anthocyanins, respectively [50]. The comparative analysis shows that the two *Vaccinium* species have different quantitative compositions of the 15 tested anthocyanins, all at different concentrations (*p* < 0.001). It was found that in bilberry there is a predominance of all target anthocyanins, except for malvidin-3-glucoside (its concentration was 471 mg and 230 mg/100 d.w. for *V. uliginosum* subsp. *gaultherioides* and *V. myrtillus*, respectively). Malvidin derivatives represented a major percentage of the anthocyanins found in bog bilberry—approximately 50% of the total concentration of target anthocyanins. The other anthocyanins identified in *V. uliginosum* occurred as follows (from lowest to highest concentration): peonidin < cyanidin = petunidin < delphinidin. As with *V. myrtillus*, glycoside abundance was also different (70% of the total), with glucosides accounting for 70% of the total, while galactosides and arabinosides were found at very similar percentages (16% and 14%, respectively) [55].

It is known that polyphenols and anthocyanins have a strong impact on antioxidant activity—the higher their content, the more potent their free-radical-eliminating action [67,68,69,70,71]. The team of Kusznierewicz et al. analyzed the content of bioactive substances in samples of wild and bog bilberry from Poland. They determined the content of anthocyanins and polyphenols in dry and fresh samples (Table 2) [72].

The polyphenolic compounds had comparable contents. Furthermore, the antioxi-dant activity of *V. myrtillus* and *V. uliginosum* was also essentially similar. The obtained results suggested that both berries are a good dietary source of anthocyanins.

#### 3.1.3. Proanthocyanidins

**Figure 5:**
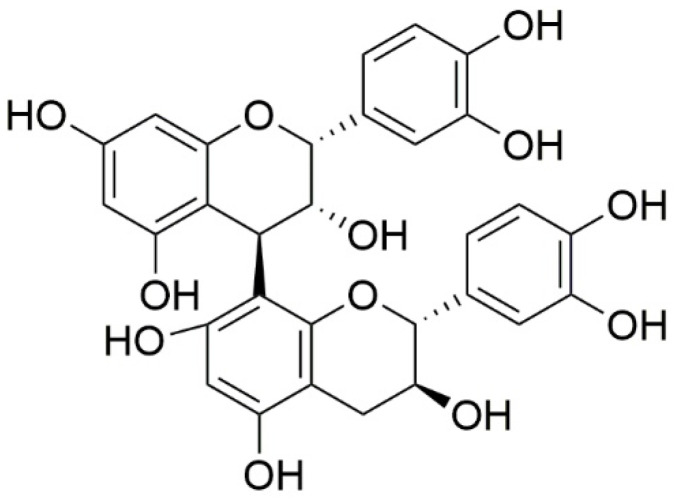
Chemical structures of procyanidin B2 contained in *V. uliginosum*.

In the dry weight (DW) of *V. uliginosum*, the main monomers and dimers of proanthocyanidins, i.e., procyanidin B2 (Figure 5), EC, phlorizin, taxifolin, gallocatechin, and EGC, were determined using a validated quantitative method. In total, the total procyanidin content was 159.4 µg/g DW, and the main monomers and dimers were EC and procyanidin B2. The content of phlorizin was 2.942 µg/g DW, and that of taxifolin was 2.807 µg/g DW. In turn, gallocatechin and EGC were identified in the tested fruits only in trace amounts [73].

#### 3.1.4. Phenolic Acids

The antioxidant effects of *V. myrtillus* fruit were shown to depend on its phenolic content. Researchers found that even very low doses of the compound produced intracellular antioxidant activity [74]. Researchers have also proven that leaves contain more phenolic compounds compared to fruits [75].

It was determined that the main phenolic acids of bog bilberry juice are protocatechuic and chlorogenic acids [56]. The total content of phenolic acids in the dry matter of bilberries is approximately 2 mg/g [73]. Similar contents of flavonoids (EC and quercetin-3-glucoside) and *p*-coumaric acid were found in *V. uliginosum* and *V. myrtillus*. It was reported that *V. uliginosum* subsp. *gaultherioides* contains twenty-fold chlorogenic acid than *V. myrtillus*. Blueberries contained about ten times more cryptochlorogenic acid (Figure 5) than bog bilberries [55]. Ellagic, gallic, *p*-coumaric, ferulic, and syringic acids constitute a higher percentage of phenolic and hydroxycinnamic acids in *V. myrtillus* fruits. Moreover, the fruit of *V. myrtillus* also contains small quantities of vanillic acid, salicylic acid, and hydroxybenzoic acid [75].

**Figure 6:**
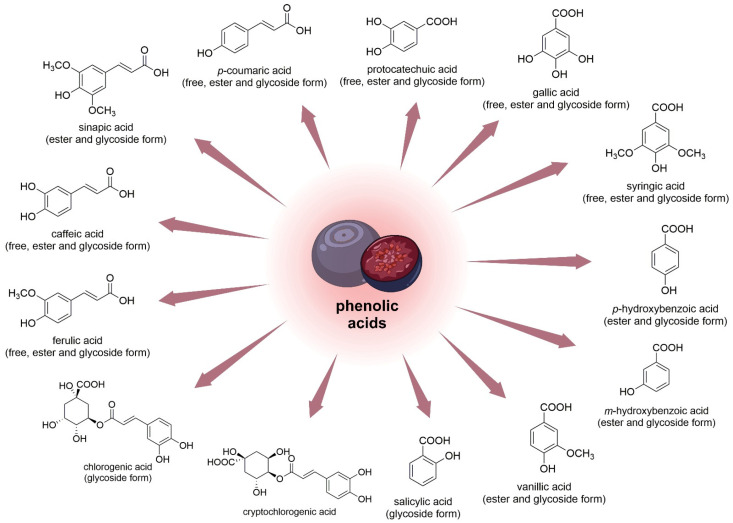
Chemical structures of phenolic acids (and their forms) that are contained in the fruits of *V. uliginosum* and *V. myrtillus* [38,55].

In one study, the quantitative composition of eleven phenolic acids (Figure 6) and seventeen anthocyanin 3-glycosides in *V. uliginosum* was identified and determined. Caffeic acid (351 and 1076 μg/100 g in free and glycoside form, respectively) and syringic acid (in ester form 3524 μg/100 g FW) were the main phenolic acids of bog bilberry. It is also worth mentioning that the content of major phenolic acids in *Vaccinium* berries seems to suggest intra- and interspecies differences [38].

### 3.2. Other Organic Acids

**Figure 7:**
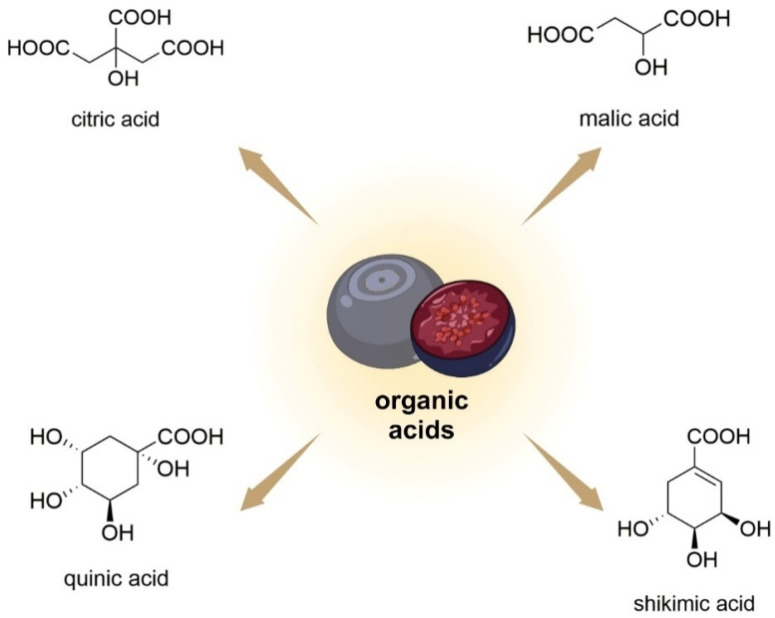
Chemical structures of organic acids that are contained in *V. uliginosum* and *V. myrtillus* fruits.

Bilberry fruits also contain simple organic acids (citric/shikimic/malic/quinic acid; Figure 7) [75]. Among the main organic acids, in terms of concentration, in *V. uliginosum* are citric acid, malic acid, and ascorbic acid, with concentrations of 172 ± 11. 21 ± 4, and 12 ± 1 mg/100 g FW, respectively [34].

### 3.3. PUFAs (Polyunsaturated Fatty Acids)

PUFAs (polyunsaturated fatty acids) are a group of exogenous fatty acids that have to be supplemented through food. This is because the human organism lacks the enzymes needed to form double bonds in the chain of fatty acids outside C-9; thus, they cannot be synthesized in our body. Fatty acids *n*-3 and *n*-6 are part of phospholipids, which are important building components of cell membranes. Importantly, the proportion of these acids in tissues depends on their dietary intake. In addition to the above, they are also essential compounds during the synthesis of many biologically active molecules, for example, prostaglandins [34].

**Figure 8:**
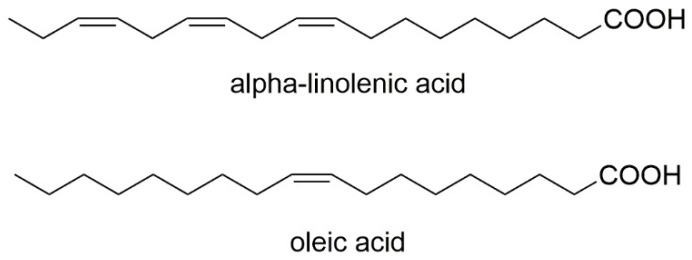
Chemical structures of fatty acids that are contained in *V. myrtillus* seed oil.

One study evaluated the chemical properties of cold-extracted native oils from *V. myrtillus* seeds to identify the qualitative composition of the fatty acids they contain and their positional distribution. It has been proven that seeds of *V. myrtillus* are abundant in PUFAs. The analysis conducted in this study showed a high α-linolenic acid (*n*-3) content in *V. myrtillus* oil, which was 28.99%. Additionally, oleic acid was detected as the predominant one in bilberry—21.02%. A very important and particularly desirable aspect of human nutrition is that vegetable oils in people’s diets should be characterized by a low *n*-6/*n*-3 acid ratio. *V. myrtillus* oils have been shown to have an *n*-3/*n*-6 ratio of 1–2, indicating that they may be beneficial in people after heart attacks and cardiac surgery [34]. Figure 8 contains the chemical formulas of the fatty acids detected in *V. myrtillus* seed oil.

### 3.4. α-Tocopherol

**Figure 9:**
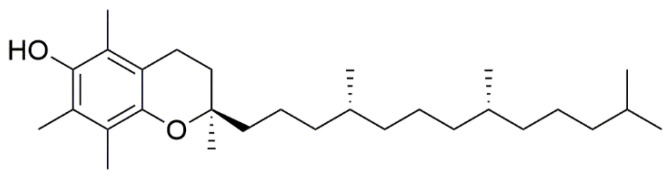
Chemical structure of α-tocopherol.

Bederska-Łojewska D. et al. found that *V. myrtillus* seed oils have higher levels of α-tocopherol (Figure 9) than commercial tocopherol-rich oils (made from soybean and corn), and 4.84 mg of vitamin E were found per 100 g of blueberry [34].

## 4. Composition and Potential of Wax

**Figure 10:**
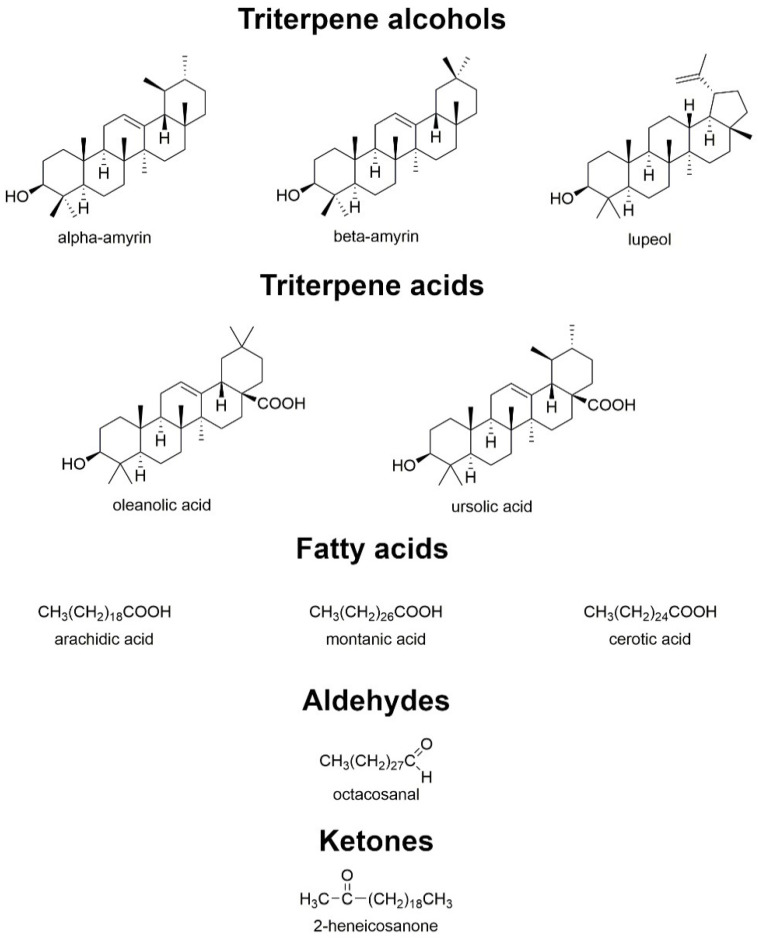
Chemical structures of compounds that are the dominant part of the wax of *V. uliginosum* and *V. myrtillus*.

The surfaces of the berries are covered by a specific waxy epidermis. Its main purpose is to protect against harmful UV radiation and prevent excessive water loss. The composition and morphology of bilberry and bog bilberry epidermal waxes were investigated. The study found that the composition of bog bilberry wax was characterized by a predominance of fatty acids, while bilberry was abundant in triterpenoids. In bilberry wax, it was discovered triterpene compounds (alcohols, i.e., lupeol, α- and β-amyrin, and acids, i.e., ursolic acid, oleanolic acid), while bog bilberry contained only triterpene acids—oleanolic acid and ursolic acid (3.1% and 1.8%, respectively) [20]. Wax content per berry increased throughout fruit development, reaching 367.6 g. Based on GC-MS analysis, triterpenoids, primary alcohols, fatty acids, compounds containing a carbonyl group (aldehydes, ketones), and alkanes predominate in the cuticular wax of fruit. Cuticular wax is generally composed of oleanolic acid as its dominant triterpenoid. As bilberry fruits develop, their wax composition changes. The proportion of triterpenoids in bilberries decreased during fruit development and the proportion of total aliphatic compounds increased [76]. The epidermal wax of bog bilberry was characterized by a predominance of fatty acids (54.8% of the total), among which arachidic acid was found in the highest amount. In bilberry, fatty acids accounted for 31.7% of the quantitative composition, and montanic acid and cerotic acid were dominant. Alkanes represented a minor part of the cuticular wax of both bilberry (2.4%) and bog bilberry (1.4%). The wax contained 10.3% aldehydes in bilberry and 7.2% bog bilberry, respectively, and octacosanal was the dominant aldehyde in both species. In turn, the fraction containing ketones was the second most quantitative component of bilberry wax (22.5% of the total), of which 2-heneicosanone was the most abundant ketone. Minor quantities of ketones were also detected in blueberry fruit wax (3.6%) [20]. The identified compounds that are the dominant part of the wax of *V. uliginosum* and *V. myrtillus* are shown in Figure 10.

## 5. The Use of Berries

### 5.1. Dermatology

Kyungae et al. investigated the photoprotective properties of the *V. uliginosum* dietary extract in hairless mice irradiated with ultraviolet B (UVB) radiation. In their study, *V. uliginosum* induced significant alterations in the water retention ability of the skin, TEWL (transepidermal water loss), and parameters related to wrinkles and skin thickness. Oral administration of *V. uliginosum* induced the upregulation of TIMP (tissue inhibitor of metalloproteinase) and antioxidant-related genes, and simultaneously reduced MMP (matrix metalloproteinase) expression. In addition, it was also responsible for a decrease in the levels of p38 protein, JNK (c-Jun *N*-terminal kinase), inflammation-activated cytokines, and UVB-induced ERK (extracellular signal-regulated kinase) phosphorylation. Additionally, *V. uliginosum* extract enriched in anthocyanins after the oral application had a positive effect on the condition and appearance of the skin after exposure to UV radiation [77]. One study investigated the potential of berry wax to block UV-B radiation. The highest SPF (sun protection factor) showed bog bilberry fruit cuticle wax. The presence of cinnamic acid and vitamin E is probably responsible for high SPF levels. Compared to lingonberry wax (supercritical fluid extraction), bilberry wax has higher levels of α-tocopherol and cinnamic acid, while bog bilberry cuticular wax is rich in cinnamic acid [20].

*V. uliginosum* extract has also been proven to be excellent for use in dietary supplements designed to take care of skin conditions, due to its abundant anthocyanin content and its anti-free radical activity [77]. One study tested the ability of anthocyanins from *V. myrtillus* to pass through the outer layer of the epidermis and prevent damage caused by the sun. A nanoberry (approximately 100 nm in diameter) exhibiting various elastic properties passed through the outer layer of the epidermis without causing harm. HaCaT cells (normal epidermal cells) in the nanoberry-containing environment remained further viable, despite being exposed to UV radiation. It was found that nanoberries can be actively taken up by cells, and the substances transported by them exhibit health-promoting effects for the skin, protect against UV radiation and facilitate wound healing [78].

Another study evaluated a topical formulation of *V. myrtillus* bilberry leaf extract and bilberry seed oil, which is a by-product of food production. In the leaf extract, the presence of chlorogenic acid was revealed as the most numerous among the phenolic acids, flavonoids—in the largest amount of isoquercetin and resveratrol—while in seed oil, the essential unsaturated fatty acids ω-3 and ω-6 were identified in a preferred proportion, close to 1. The anti-free radical potency was also assessed by wild blueberry extract and seed oil. In a study involving healthy volunteers, the impact of topically applied oil-in-water (o/w) creams containing these wild bilberry isolates was studied. Using wild bilberry isolates as active ingredients, o/w cream was found to improve the skin barrier function and tolerability, as well as retain the pH value of the skin and significantly increase stratum corneum hydration. A cream formulated with wild bilberry isolates as biologically active compounds may be used to treat skin disorders associated with oxidative stress and/or dry skin in addition to their good sensory properties [79].

### 5.2. Ophthalmology

*V. uliginosum* extract contains numerous antioxidant compounds, the supplementation of which has a proven effect in alleviating the symptoms of dry eye [80,81,82]. In a randomized, double-blind study, participants were divided into two groups—the study group (29 subjects) and the control group (30 subjects). The study group received oral tablets with *V. uliginosum extract* (1000 mg/day, total polyphenol content 9.1 mg/g) and the control group took a placebo (lactose). The duration of the trial was 4 weeks. The study proved that taking *V. uliginosum* extract significantly alleviated the visual discomfort caused by working in front of a computer and tablet screen. In the study group, they suggested significant improvement, including questions about “tired eyes” (*p* = 0.001), “irritated eyes” (*p* = 0.010), “eye pain” (*p* = 0.038), “watery eyes” (*p* = 0.005), “dry eyes” (*p* = 0.003), “visual discomfort” (*p* = 0.018), and “blurred vision” (*p* = 0.035). The control group showed improvement only for “tired eyes” (*p* = 0.002) and “irritated eyes” (*p* = 0.033) [83].

Researchers demonstrated that *V. uliginosum* extracts protected retinal cells from light-induced damage [84]. One study investigated the protective action of anthocyanins isolated from *V. uliginosum* on retinal cells against damage caused by microwave radiation. The study determined the cell apoptosis index (AI), malondialdehyde (MDA), glutathione (GSH), and SOD activity. The mRNA expression levels of HO-1(heme oxygenase-1) and Nrf2 (nuclear factor 2-related factor 2) proteins were also investigated. The rate of cell apoptosis was reported to be notably elevated in the control group in comparison to the *V. uliginosum* treatment group, and the decrease in AI was correlated with dose. MDA and GSH in the study group were also lower and SOD activity was significantly higher. The expression of mRNA Nrf2 and HO-1 proteins increased marginally after irradiation and rose in the treated group. Based on this study, it was revealed that anthocyanins extracted from *V. uliginosum* have a stabilizing effect on the cell membrane, reduce apoptosis, and alleviate oxidative stress-induced damage to mouse retinal photoreceptor cells. Activation of the Nrf2/HO-1 signaling pathway and induction of HO-1 enzyme expression may be responsible for the above mechanism [85].

In a study carried out by Choi et al., the effect of *V.uliginosum* L. on cataract formation in Sprague–Dawley (SD) rat pups was assessed. The morphological analysis of the lens showed that the use of extract inhibits m-calpain-mediated proteolysis, PARP (Poly (ADP)-ribose polymerase) cleavage, and oxidative stress in the lens. *V.uliginosum* suppressed cataract development in a dose-dependent manner by preserving the expression of Nrf-2/HO-1 pathway proteins, maintaining cellular antioxidant protection, and inhibiting the insolubility of soluble proteins, including crystallins [86].

The team of Yoon SM et al. evaluated the effectiveness of *V. uliginosum* L. (VU) and its fractions in the prevention of AMD (age-related macular degeneration) development in human RPE (retinal pigment epithelium) cells exposed to blue light (ARPE-19 cells). During the viability assay, ARPE-19 cells were treated simultaneously with A2E (*N*-retinyl-*N*-retinylidene ethanolamine) and VU (or a fraction thereof). It was observed that after the exposure of ARPE-19 cells to A2E and blue light, the total protein level declined by 55%, which implies a reduced cell survival by blue light-induced A2E photooxidation. In turn, the treatment of the tested cells with VU (concentration of 500 µg/mL) resulted in a 28.7% reduction in the percentage of dead cells (in comparison with the A2E-BL group) after exposure to blue light. A fast atom bombardment mass spectrometry (FAB-MS; cell-free system) analysis revealed that anthocyanin and polyphenol decreased the A2E oxidation peak. The results of this study show that anthocyanin and polyphenol efficiently suppress the A2E oxidation induced by blue light exposure.VU extract, as well as its fractions, have a prophylactic action on RPE cell damage and AMD induced by blue light exposure [87,88,89]. FH (fraction of *Vaccinium uliginosum* L.) suppressed PMA-induced activity of AP1 and NF-κB proteins in a dose-dependent manner in A549 cells. In the course of the study, it was found that treatment using FH induced a reduction in *CXCL2* and *RASD1* gene expression and exerted antioxidant activity (↓ROS) in comparison with A2E-laden RPE cells illuminated with blue light [89].

The team of Lee BL et al. conducted a study in which RPEs were tested for their resistance to damage from blue light using polyphenol-containing extracts. According to their findings, *V. uliginosum* extract and eluted fractions may be possibly used as a therapy strategy for age-related macular degeneration [88].

Additionally, for studies using *V. myrtillus* and/or *V. uliginosum*, we highlight two clinical trials in the field of ophthalmology investigating the impact of extracts from these plants on the organ of vision. The first of them (NCT number: NCT04063644) was designed to study the efficacy of eye drops containing *V. myrtillus* in their composition in the course of dry eye syndrome and their effect on improving visual acuity [90]. Meanwhile, the second trial (NCT number: NCT02641470) evaluated the preventive action of *V. uliginosum* extract against asthenopia induced by spending time in front of a computer monitor. Pills containing 1000 mg/day of *V. uliginosum* extract (DA9301) or a placebo were given orally to participants for 4 weeks, and then the results were assessed using an appropriate questionnaire [91].

### 5.3. Gynecology

In the study, Ozlem et al. *V. myrtillus* prevented I/R (ischemia-reperfusion) injury in ovarian tissue. In the control group (without receiving medication) that underwent 1-h ischemia and 2-h reperfusion ovary, malondialdehyde (MDA) levels were notably elevated, and enzymatic activities of CAT and SOD were markedly decreased compared to groups with the same damage but receiving a single dose of 200 mg/kg *V. myrtillus*. Moreover, in a histopathologic examination, the damage to ovarian tissues was significantly greater and had a significantly higher DNA damage and apoptosis in the group without a dose of *V. myrtillus*. As part of a gynecology practice, ovarian torsion is diagnosed and subsequently treated medically. This treatment, unfortunately, has limited protective effects if ovarian torsion occurs [92].

The treatment of ovarian cancer is a tremendous challenge for clinicians because it is often chemoresistant. In one study, the antiproliferative activity of 36% anthocyanin-enriched *V. myrtillus* extract in ovarian cancer cells that were sensitive and resistant to conventional chemotherapeutic treatment was studied. The tested mixture consisted of delphinidin, cyanidin, malvidin, peonidin and petunidin (in the proportion 33:28:16:16:7, respectively), which were in glycosylated forms. The above results lead to the conclusion that this mixture can sensitize chemotherapy-resistant ovarian cancer cells and reduce, in a dose-dependent manner, the effective dose of cisplatin required for a therapeutic response. Additionally, this study provides some evidence suggesting the possible benefits of combining conventionally used paclitaxel with a naturally derived product such as berry anthocyanidins in the treatment of chemo-resistant ovarian tumors [93].

### 5.4. Diabetology

Bilberry polyphenols have been shown to positively impact metabolic health in several animal studies, but studies are often conducted with pharmacological doses that have little nutritional relevance [94,95,96].

Recent studies show that protein tyrosine phosphatase 1B (PTP1B) and α-glycosidase have demonstrated effectiveness in controlling type 2 diabetes. Alternative treatment methods for this disease may include combined therapeutic strategies. By inhibiting these enzymes, polysaccharides may be absorbed and disintegrated more slowly, and blood glucose levels may rise more quickly post-prandial. In the insulin signaling pathway, the overexpression of PTP1B can inhibit insulin expression as a negative regulator [97]. The anthocyanins from *V. uliginosum* are the most potent inhibitors of PTP1B (IC_50_= 3.06 ± 0.02 μg/mL). Based on the molecular docking research, cyanidin-3-O-glucoside had the lowest affinity for inhibiting PTP1B versus any other skeleton, whereas cyanidin-3-O-glucoside exhibited the highest affinity for inhibiting PTP1B (binding energy (E_B_) = −7.8 kcal/mol), interacting with its two binding sites [98].

A randomized, double-blind, placebo-controlled crossover study was conducted on 20 patients. The treatment lasted 4 weeks. The study design involved receiving two capsules twice a day and was divided into two arms—the placebo group (starch) and the intervention group (*V. myrtillus*; 1.4 g/day of anthocyanin extract). After the treatment period, there was a 6-week procedure for the washout of the drug from the body, after which the patients’ treatment regimen was switched. The study enrolled patients diagnosed with type 2 diabetes treated with hypoglycemic drugs with a BMI (body mass index) > 23 kg/m^2^ and no evidence of cardiovascular disease. In the group that supplemented with bilberry, there was a tendency to reduce fasting glucose and HBA1c levels; in the placebo group, this relationship was not noticed. This may indicate that higher doses or longer duration of treatment may favor glycemic control [99]. In another study, aqueous and methanol extracts were proven to be effective α-glucosidase inhibitors. Through the inhibition of α-glucosidase by *V. myrtillus* extracts, patients with type 2 diabetes can control their glycemic level through diet [100].

In a study by Xingguo Li et al., a new polysaccharide fraction (VUP-1) from *V. uliginosum* L. was obtained using pressurized water extraction, and purified using a polyamide resin column and column chromatography.VUP-1, from the fruits of *V. uliginosum* L., is a heteropolysaccharide consisting of galacturonic acid, galactose, glucose, mannose, and arabinose, with an MW (molecular weight) of 4.98 × 10^4^ kDa. The inhibition of α-amylase by VUP-1 is moderate and characterized by high antioxidant activities. Furthermore, VUP-1 inhibits dicarbonyl compound formation. The results indicate that VUP-1 had an uptake effect on free radicals (OH and DPPH) in a dose-dependent manner (*p* < 0.05). The results of this study indicate that polysaccharides from *V. uliginosum* L. could potentially be used as oral hypoglycemic agents [101].

Kim J et al. conducted a study to determine whether *V. myrtillus* bilberry helps prevent diabetes-induced retinal vascular dysfunction in vivo. Streptozotocin-induced diabetic rats were orally fed *V. myrtillus* extract (VME; 100 mg/kg) for 6 weeks. Diabetic rats undergoing treatment with VME exhibited a notable decline in fluorescein leakage in fluorescein-dextran angiography. VME treatment reduced specific indicators of diabetic retinopathy, such as the degradation of OCLN (occludin), ZO-1 (zonula occludens-1), CLDN5 (claudin-5), and VEGF (retinal vascular endothelial growth factor) expression in diabetic rats. It has been proven that VME can prevent or retard the development of early diabetic retinopathy [102].

In a study by Pemmari T et al. conducted in obese mice induced by a high-fat diet, the effects of dried blueberry powder on parameters such as body weight increase, systemic inflammation, glucose/lipid metabolism, and changes in gene expression in liver and adipose tissue were investigated. Blueberry supplementation prevented the rise of alanine transaminase (ALT; a marker of liver damage) and many proteins involved in the inflammatory response, such as serum amyloid A (SAA), CXC chemokine ligand 14 (CXCL14) and monocyte chemoattractant protein-1 (MCP1) induced by the high-fat diet. As a result of blueberry supplementation, serum insulin, glucose, and cholesterol concentrations were partly reduced, systemic and hepatic inflammation was suppressed, and undesirable changes in glucose/lipid metabolism were retarded. Consequently, blueberry supplementation appeared to support a healthier metabolic phenotype during obesity development [103].

Type 2 diabetes is very often not a single disease entity in the people suffering from it. Most patients are diagnosed with multiple coexisting factors associated with the development of this disease, among others, including impaired glucose tolerance or dyslipidemia and associated further atherosclerosis [104]. Table 3 shows clinical trials investigating the effects of *V. myrtillus,* not only on type 2 diabetes but also in the above-mentioned metabolic disorders.

### 5.5. Cardiology

**Figure 11:**
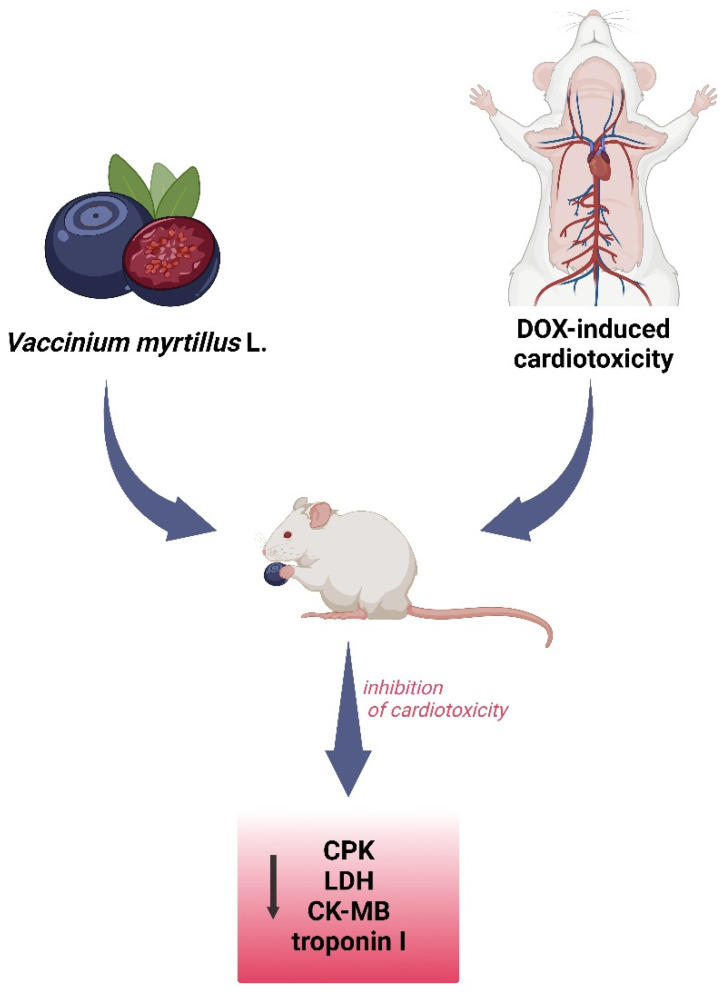
Cardioprotective effect of *V. myrtillus* against doxorubicin toxicity. DOX—doxorubicin, CPK—creatine phosphokinase, CK-MB—creatine kinase-myocardial band, and LDH—lactate dehydrogenase.

In the clinical picture of cardiovascular disease, high levels of circulating microvesicles (MVs) and an increased risk of atherosclerosis can be distinguished. A study available in the literature evaluated the effect of bilberry extract (BE) on participants’ MV levels and its impact on endothelial vesicles in vitro. Patients with myocardial infarction were supplemented with BE for eight weeks. The findings showed that BE supplementation positively affected the MV profile in participants’ blood and decreased extracellular vesicle release via a P2X7 receptor-dependent mechanism. The cardioprotective effect of blueberries has been proven [109]. The antioxidant properties of *V. myrtillus* may partly explain its ability to protect rats from doxorubicin (DOX)-induced cardiotoxicity. DOX-induced elevations of lactate dehydrogenase (LDH), creatine phosphokinase (CPK), creatine kinase-myocardial band (CK-MB), and troponin I (TNI) activity in serum were significantly inhibited by bilberries. The treatment with *V. myrtillus* reduced the severity of histological lesions in rat tissue sections (Figure 11) [110].

Habanova M et al. conducted a study of 25 women and 11 men who ate frozen blueberries (3 times a week, 150 g each) for 6 weeks. The consumption of blueberries resulted in decreased parameters of glucose (*p* = 0.005), γ-glutamyltransferase (*p* = 0.046), albumin (*p* = 0.001), TG (triglyceride; *p* = 0.001), total cholesterol (TC; *p* = 0.017), LDL-C (low-density lipoprotein cholesterol; *p* = 0.0347), and elevated HDL-C (high-density lipoprotein cholesterol; *p* = 0.044). Additionally, in the male population, a positive influence of bilberry consumption on albumin (*p* = 0.028), γ-glutamyltransferase (*p* = 0.013), aspartate aminotransferase (*p* = 0.012), glucose (*p* = 0.015), TC (*p* = 0.004), and HDL-C (*p* = 0.009) was observed, with a rise in LDL-C (*p* = 0.007) also noted. The study showed that the systematic consumption of blueberries may reduce the risk of cardiovascular disease by lowering the levels of TG and LDL-C with a simultaneous increase in HDL-C [111]. In another study of 32 adult rats supplemented with *V. myrtillus* powder (2 g/day) for four weeks, a significant improvement in diabetic dyslipidemia was observed by lowering TC, TG, LDL-C, and VLDL-C in plasma [112]. The studies show that regularly consuming frozen bilberries for even a short period can improve humans’ lipid profile [111].

In one study conducted on rats, it was proven that *V. myrtillus* bilberry anthocyanin (BA) notably enhanced total antioxidant ability, total CAT and SOD activity, leading to reduced levels of glycated serum protein (GSP), MDA, TG, TC LDL-C and lower Castelli Index I and II values (TC/HDL-C and LDL-C/HDL-C, respectively) [113].

### 5.6. Antimicrobial Activity

The team of Benassai E et al., during green synthesis and using aqueous extracts of bilberry (*Vaccinium myrtillus* L.) and bog bilberry fruit (*Vaccinium uliginosum* L. subsp. *gaultherioides*), obtained mixtures that contained copper nanoparticles (Cu-NPs) in their composition and then underwent microbiological investigation. The obtained mixtures were characterized by potent and extensive antimicrobial activity (fungi, Gram-negative and positive bacteria), and their activity was stronger in most cases compared to equivalent concentrations of copper salts [114]. In one study, the authors focused on evaluating the antibacterial activity of *V. uliginosum* extract and its fractions against Gram-negative (*Vibrio parahaemolyticus*, *Salmonella enteritidis*) and Gram-positive (*Staphylococcus aureus*, *Listeria monocytogenes*) bacteria. The crude extract (BBE) of wild blueberry (*Vaccinium uliginosum*) was achieved by extraction with methanol, and the F1, F2, and F3 fractions (sugars/acids, phenols, and anthocyanins/proanthocyanidins, respectively) were isolated. The F3 strain exhibited the most potent antibacterial effect compared to the other examined strains, and then the F2, F1 and BBE strains. Gram-negative bacteria, compared to Gram-positive bacteria, exhibited greater sensitivity to all fractions, with the sensitivity of the tested species, as follows (least→most sensitive): *S. aureus*→*L. monocytogenes*→*S. enteritidis* →*V. parahaemolyticus*. The received results indicate that the investigated blueberry fractions (in particular, F3) suppress the growth of bacteria, whose route of infection is food, as a consequence of damage to their cytoplasmic membrane. This information could be used to create new natural preservatives to protect food from pathogenic microorganisms in the future [115].

Different concentrations of anthocyanins from *V. uliginosum* were applied to four types of pathogens to test their antibacterial properties. A positive correlation was found between anthocyanin concentration and antimicrobial activity. Overall, significant antimicrobial activity against *S. enteritidis, V. parahaemolyticus, L. monocytogenes*, and *St. aureus* was observed when anthocyanins were used at 0.53 mg/mL. Anthocyanins at 0.26 mg/mL completely suppressed *S. aureus*, and reduced *L. monocytogenes* by 3.27 log and *S. enteritidis* by 1.07 log. The presence of anthocyanins also increased protein efflux from *L. monocytogenes*, *S. enteritidis*, *S. aureus*, and *V. parahaemolyticus* across damaged membranes [101].

The study by Satoh, Yutaroh, and Kazuyuki Ishihara aimed to identify the antibacterial compounds present in *V. myrtillus* that inhibited periodontopathic bacteria. Oil/water separation was used to extract the acetone-soluble fraction of *V. myrtillus*. In the following step, the extract was purified by chromatography using silica gel. The total extract had an MIC (minimum inhibitory concentration) of 500 g/mL against *Porphyromonas gingivalis*. An antibacterial fraction called NU4-TDC was found to be effective against *P. gingivalis*. This product had MICs above 62.5 μg/mL for *Streptococcus mutans*, 26.0 μg/mL for *P. gingivalis*, 59.1 μg/mL for *Fusobacterium nucleatum*, and 45.1 μg/mL for *Prevotella intermedia*. Based on the above-mentioned research, it can be concluded that bilberry extract has antimicrobial properties. The semi-purified fraction (NU4-TDC) also demonstrated antimicrobial activity when tested against *P. intermedia*, *P. gingivalis*, and *F. nucleatum* [116].

In another investigation, a team of researchers undertook to determine the antifungal effects and content of particular compounds in essential oils from *Vaccinium myrtillus*. It was found that the essential oil extracted from this plant consists of 41.07% 1,8-cineole, 12.72% β-linalool, 12.17% α-pinene, and 6.48% myrtenol. *V. myrtillus* essential oil suppressed mycelial growth in *Alternaria solani, Verticillium dahlia Kleb, Sclerotinia sclerotiorum* (Lib.), and *Fusarium oxysporumf*. sp. *radicis-lycopersici* (Sacc.) W.C. Synder & H.N. Hans (FORL) by 100%, 57.91%, 61.38%, and 80.36% respectively. The findings of this research revealed that *V. myrtillus* essential oil exhibits potent antifungal properties [117].

### 5.7. Oncology

Colorectal cancer is a malignant process that develops in the final part of the gastrointestinal tract and is one of the most lethal types of cancer globally. Lippert E et al. assessed the influence of an anthocyanin-rich blueberry extract on colorectal tumor progression and growth after administration of azoxymethane (AOM)/sodium dextran sulfate (DSS) using an in vivo mouse model. Mice fed 10% anthocyanins exhibited markedly (*p* < 0.004) less reduction in colon length compared to the control group, providing evidence of reduced inflammation. Moreover, mice in the control group and those receiving 1% anthocyanins showed a higher average number of tumors when compared to individuals receiving 10% anthocyanin-rich extract. Anthocyanins prevented the initiation and progression of colorectal cancer in Balb/c mice exposed to AOM/DSS [118].

In another study, the impact of a standardized *V. myrtillus* extract on human colorectal adenocarcinoma cells (Caco-2) was investigated. The tested extract contained anthocyanins 237.9 ± 17.1 mg CGE (cyanidin-3-glucoside equivalent)/g, phenols 338.5 ± 28.0 mg GAE (gallic acid equivalent)/g, and flavonoids 735.4 ± 18.2 mg QE (quercetin equivalent)/g. It was measured whether *V. myrtillus* may modify the expression of genes related to cholesterol biosynthesis. One of the main transcription factors for cholesterol uptake and biosynthesis is SREBP2 (sterol regulatory element-binding protein 2). It regulates LDLR (LDL receptor) and HMGR (3-hydroxy-3-methylglutaryl coenzyme A reductase). After treatment with blueberries, the abundance of SREBP2 and HMGR mRNA decreased in a statistically significant way. In contrast, LDLR expression in Caco-2 cells increased 2-fold. In conclusion, the *V. myrtillus* extract modulated genes that are involved in cholesterol metabolism in the intestine [119].

Mauramo M. et al. performed a study to investigate the influence of bilberry powder on OSCC (oral squamous cell carcinoma) cells using in vitro/in vivo assays. In a study comparing 0.01 mg/mL cetuximab with 0, 1, 10, and 25 mg/mL powder obtained from whole berries, invasion, proliferation, migration, and viability were assessed in OSCC cells (HSC-3). The in vitro study revealed that bilberry powder exhibited antiproliferative activity and inhibited the migration and invasion process, while the suppression of tumor progression was observed in the in vivo investigation. The inhibitory activity of the tested powder intensified with rising concentrations and was more pronounced in cancer cells when compared to normal cells. When compared with cetuximab, bilberry powder exhibited comparable or even more potent activity in a dose-dependent manner [120].

It was tested whether the exosomal *V. myrtillus* bilberry’s anthocyanins and their aglycones anthocyanidins (ExoAnthos) would increase the therapeutic effectiveness over free Anthos against A549 lung cancer cells. The antiproliferative activity of Anthos and ExoAnthos was determined using subcutaneous lung cancer xenografts in athymic nude mice, and then it was compared with exosomes alone. Regardless of the tumor cell type, ExoAnthos exerted a notably stronger dose-dependent antiproliferative effect compared to free Anthos. The greater efficacy of exosomal Anthos is partly due to their intrinsic activity, which is an ‘add-on’ effect not observed with traditional systems [121].

Li J et al. studied the effect of BA on healthy ageing in 12-month-old ageing female rats. The findings suggest that the intake of a medium dose of BA (MBA) markedly elevated relative the liver weight by 7.34% compared to an ageing control group. In feces, MBA decreased bacterial enzyme activity and increased short-chain fatty acids. The results of the Western blot analysis indicated an increased expression of ZO-1, OCLN, and autophagy-related proteins (ATP6 V0C (bafilomycin A1-binding subunit of vacuolar ATPase), ATG4D (autophagy-related 4D cysteine peptidase), and CTSB (cathepsin B)) in ageing rats. MBA induced AMPK (5′AMP-activated protein kinase) and FOXO3a (forkhead box O3) phosphorylation and inhibited mTOR (mammalian target of rapamycin) phosphorylation, indicating that blueberry anthocyanin induced autophagy through the AMPK/mTOR signaling pathway. Furthermore, the activation of autophagy additionally promoted the ability to counteract the effects of oxidative stress and enhanced the intestinal epithelial barrier function in ageing female rats [113].

Kausar H. et al. carried out a study in which they found that combining suboptimal equimolar concentrations of anthocyanidins from *V. myrtillus* with marginal effects on the viability of normal cells synergistically suppressed the proliferation of two aggressive NSCLC (non-small cell lung cancer) cell lines. A mixture of anthocyanidins significantly induced the apoptotic process and cell cycle arrest in the G_2_/M phase and suppressed the cancer cell migration and invasion process compared to a single anthocyanidin. The improved efficacy of the combinatorial treatment was probably a result of the enhanced cleavage of the apoptosis mediators Bcl2 and PARP, its effect on the oncogenic Wnt/Notch pathway and its downstream signaling molecules (β-catenin, c-myc, cyclin B1 and D1, MMP9, pERK, and VEGF), and elevated suppression of NF-κB activation via the TNF-α-dependent pathway. H1299 xenografts were significantly inhibited in nude mice by both the native mixture of anthocyanidins in bilberries (0.5 mg per subject) and delphinidin (1.5 per subject) [122].

Besides in vitro and in vivo animal research, one clinical trial can be found (NCT number: NCT01674374, phase 2) which focused on the treatment of mucositis induced by radiotherapy and chemotherapy for HNSCC (head and neck squamous cell carcinoma). Immediately after the appearance of symptoms of inflammation and ulceration present in the oral cavity, patients were given granules, which in their composition contained, among other things, *V. myrtillus* extract or a placebo. The intake of the preparation lasted up to 4 weeks after the end of radiotherapy, with the duration of therapy not to exceed 11 weeks. After this period, participants were asked to assess their quality of life and fill out an appropriate questionnaire [123].

## 6. Side Effects

According to the American Herbal Products Association, bilberries have been recognized as a class 1 herb, and are therefore considered safe to consume when used in appropriate amounts [124]. In clinical trials, no disturbing side effects were noted. Interactions with other drugs have also not been demonstrated [97,125,126,127]. It is important to monitor patients for bleeding disorders, due to the antiplatelet effect of *V. myrtillus*; this applies to patients who take antiplatelet drugs and additionally supplement with blueberry extract for a longer period.

## 7. Conclusions

Health benefits, biological activity, and the composition of blueberry and bog bilberry were reviewed. Blueberries contain large amounts of anthocyanins, which are known for their potent biological activity as antioxidants and, according to studies, may be involved in the prophylaxis of cancer or other diseases, including those of metabolic origin—these reports indicate the incredible health potential of blueberries. Because of their antioxidant, anti-inflammatory, anti-cancer, and apoptosis-reducing activity, both bog bilberries and bilberries can be used interchangeably as a dietary supplement with anti-free radical action, in the prevention of cancer diseases and cataracts, or as a component of sunscreen preparations. The composition of both blueberries was analyzed, and they contain many bioactive compounds (including antioxidants and flavonols) with a beneficial effect on health.

*Vaccinium uliginosum* has not yet been as well researched in terms of both the content of metabolites and its biological activity as other *Vaccinium* species. There are many studies indicating the prevention and possible treatment of cancer with products derived from *V. uliginosum* blueberries, but they are still little confirmed.

## Figures and Tables

**Table 1:** Method of characterization of some polyphenol compounds in berries of *Vaccinium* genus.

Polyphenol Compounds	Method of Characterization	References
delphinidin-3-*O*-galactoside malvidin-3-*O*-galactoside malvidin-3-*O*-arabinoside delphinidin-3-*O*-arabinoside	CIELAB HPLC-DAD	[46]
delphinidin 3-glucoside cyanidin 3-glucoside petunidin 3-glucoside delphinidin 3-glucoside	HPLC-DAD	[47]
chlorogenic acid quercetin-3-*O*-galactoside quercetin-3-*O*-glucuronide delphinidin-3-*O*-galactoside delphinidin-3-*O*-glucoside cyanidin-3-*O*-galactoside petunidin-3-*O*-glucoside	HPLC-UV/DAD HPLC-ESI-MS MS	[48]
delphinidin 3-*O*-glucoside malvidin 3-*O*-glucoside myricetin 3-*O*-hexoside quercetin 3-*O*-galactoside	HPLC-DAD HPLC-ESI-MS	[49]
cyanidin-3-*O*-glucoside cyanidin-3-*O*-rutinoside catechin quercetin-3-*O*-galactoside quercetin-3-*O*-arabinoside myricetin 3-*O*-hexose	HPLC-FT-ICR MS/MS	[50]
gallic acid vanillic acid ferulic acid caffeic acid *p*-coumaric acid quercetin	HPLC	[51]
(–)-epicatechin kaempferol derivative chlorogenic acid ellagic acid	HPLC	[52]
glycosides of quercetin myricetin kaempferol isorhamnetin syringetin laricitrin	HPLC–MS	[53]

The colors used in Table 1: red—phenolic acids, green—flavonols, and violet—anthocyanins.

**Table 2:** The total content of anthocyanins and other polyphenolic compounds in dry and fresh weight of Polish *V. myrtillus* and *V. uliginosum*.

	Dry Samples		Fresh Samples	
	Total Anthocyanins Content (mg/g)	Total Phenolics Content (mg/g)	Total Anthocyanins Content (mg/g)	Total Phenolics Content (mg/g)
*V. myrtillus*	21.8 ± 0.1	26.6 ± 0.1	19.4 ± 0.1	23.7 ± 0.1
*V. uliginosum*	14.3 ± 0.3	21.1 ± 0.3	12.4 ± 0.2	18.2 ± 0.2

**Table 3:** Clinical trials evaluating the effects of *V. myrtillus* on metabolic disorders. This table is compiled from the information available at https://www.clinicaltrials.gov/, accessed on 9 August 2023.

NCT Number	Study Title	Clinical Trial Status	Study Design	Condition	References
NCT01860547	The Effect of the Bioactives of Sea Buckthorn and Bilberry on the Risk of Metabolic Diseases	Not applicable	Randomized, open-label, crossover assignment	Type 2 Diabetes Atherosclerosis	[105]
NCT01414647	The Effect of Diet Rich in Nordic Berries on Gut Microbiota, Glucose and Lipid Metabolism and Metabolism on Fenolic Compounds	Not applicable	Randomized, open-label, crossover assignment	Metabolic Syndrome Impaired Glucose Tolerance Low-grade Inflammation Dyslipidemia	[106]
NCT03415503	Anthocyanin Supplementation Improves Blood Lipids in a Dose-response Manner in Subjects with Dyslipidemia	Phase 3	Randomized, double-blind (participant, investigator), parallel assignment	Dyslipidemia	[107]
NCT04054284	Safety and Efficacy of a Complex Herbal Tea Mixture in Type 2 Diabetics	Not applicable	Randomized, quadruple -blind (participant, care provider, investigator, outcomes assessor), parallel assignment	Type 2 Diabetes	[108]

## Data Availability

No new data were created or analyzed in this study. Data sharing is not applicable to this article.
